# Evaluating plasticity of rejuvenated metallic glasses by effective enthalpy

**DOI:** 10.1016/j.fmre.2025.03.008

**Published:** 2025-03-25

**Authors:** Cheng Yang, Hong-Bo Zhou, Jun Duan, Song-Lin Cai, Gan Ding, Bing-Bing Zhang, Cai-Juan Shi, Lan-Hong Dai, Gerhard Wilde, Min-Qiang Jiang

**Affiliations:** a State Key Laboratory of Nonlinear Mechanics, Institute of Mechanics, Chinese Academy of Sciences, Beijing 100190, China; b School of Engineering Science, University of Chinese Academy of Sciences, Beijing 101408, China; c Institute of Materials Physics, University of Münster, Münster 48149, Germany; d Beijing Synchrotron Radiation Facility, Institute of High Energy Physics, Chinese Academy of Sciences, Beijing 100049, China

**Keywords:** Metallic glasses, Structural rejuvenation, Effective enthalpy, Plastic deformability, Structural heterogeneity

## Abstract

Structural rejuvenation is a promising strategy to enhance the plasticity of metallic glasses, but quantitatively evaluating the rejuvenation-deformation relationship is challenging. In this work, we systematically investigate the compressive plasticity of a typical Zr-based metallic glass which is rejuvenated by elastostatic compression or cycled twists. Meanwhile, the thermodynamics and structural properties are carefully characterized for these rejuvenated glasses. An obvious gap in the plastic strain *ε*_p_ is observed between two sets of the rejuvenated glasses, although they have very close exothermic enthalpy ∆*H*_exo_ before glass transition. This plasticity gap is also reflected by the different modes of shear banding: straight for the elastostatic compression but deflected for the cycled twists. We use the concept of effective enthalpy Δ*H*_eff_ and find a linear relationship between Δ*H*_eff_ and *ε*_p_ for all rejuvenated glasses. This implies that the plastic deformability of glasses is determined not only by the existing free volume evaluated by ∆*H*_exo_ but also by the creating free volume required to enter the supercooled liquid state, as evaluated by the endothermic glass transition overshoot. Synchrotron high-energy X-ray diffraction reveals that the larger *ε*_p_ in glasses with higher Δ*H*_eff_ arises from the enhanced structural heterogeneity caused by the decoupling of medium-range orders.

## Graphical abstract

**Figure:**
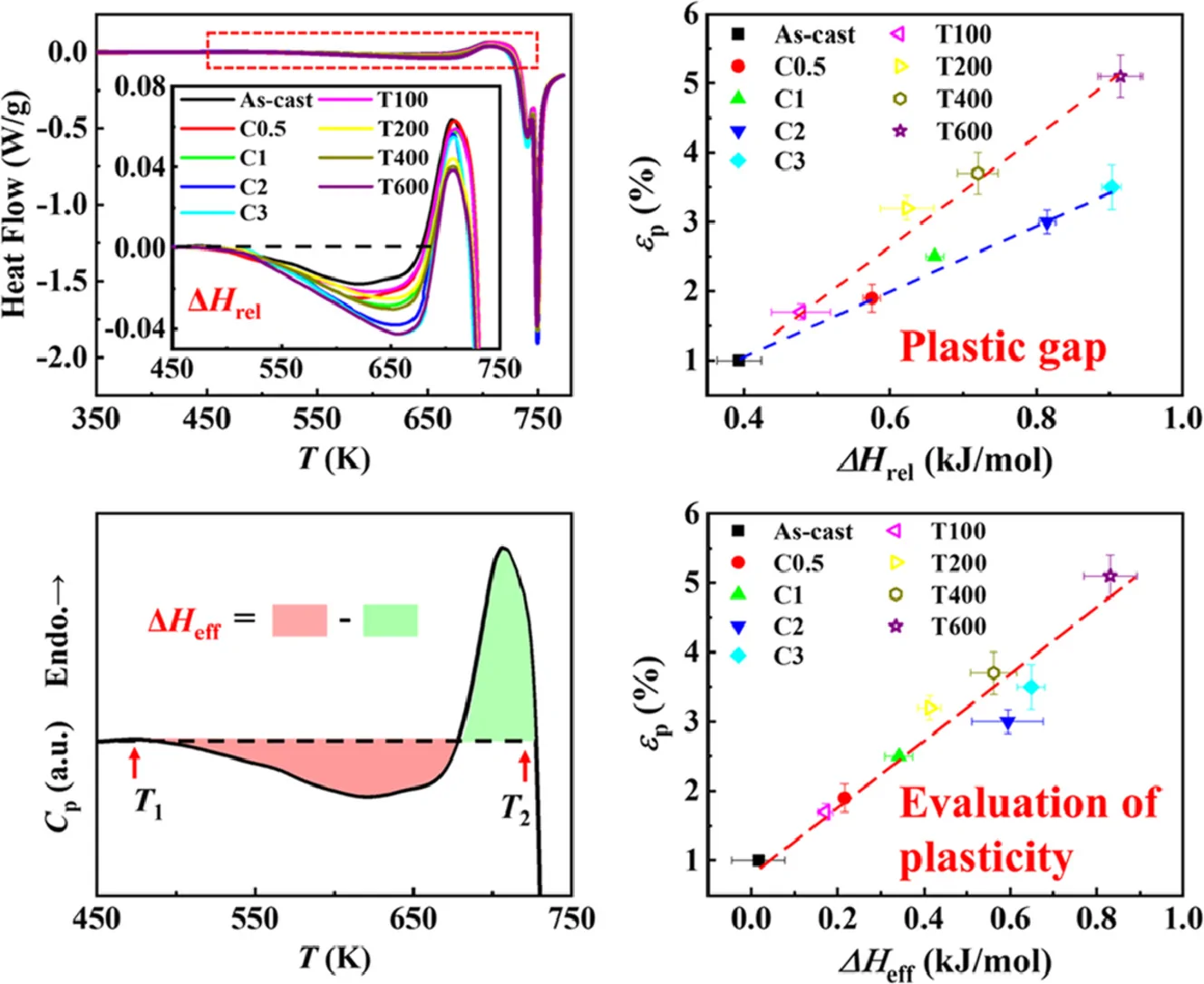

## 1 Introduction

Despite the fascinating characteristics such as high strength and elasticity [[1], [2], [3], [4]], metallic glasses (MGs) usually exhibit limited room-temperature plasticity which greatly impedes their practical applications. Structural rejuvenation, as an inverse process of physical aging, is commonly employed to improve the plasticity of MGs. Typical rejuvenation protocols include thermal cycling [[5], [6], [7]], mechanical loading [[8], [9], [10]], thermomechanical creep [[11], [12], [13]], etc. The plastic strain *ε*_p_ of rejuvenated glasses usually increases with increasing exothermic enthalpy ∆*H*_exo_ before the glass transition [14,15]. However, Meng et al. [16] found that this positive relationship between *ε*_p_ and ∆*H*_exo_ is not always satisfied in the rejuvenated MGs with different thermal histories. This implies that the ∆*H*_exo_ as a system-averaged atomic spacing or free volume cannot fully determine the macroscopic plasticity. Jiang et al. [17] analyzed the so-called Poisson's ratio criterion for MGs’ plasticity from short-range-order (SRO) structures. They highlighted that a perfect matching of dense and disordered SROs facilitates plasticity. A proper physical variable beyond SRO is necessary to evaluate the global plasticity of rejuvenated MGs.

Using synchrotron high-energy X-ray diffraction (XRD), Dmowski and co-workers [12,[18], [19], [20]] have revealed that MGs rejuvenate via complex structural rearrangements from SRO to nanometer length scales. With shock compressions, Ding et al. [8] indeed revealed that structural rejuvenation involves nanoscale shear-transformation (ST)-type rearrangements that underlie an increase of ∆*H*_exo_ or atomic free volume. After investigating the rejuvenation behavior of as-cast and pre-annealed MGs, Zhou et al. [21] found that the fictive temperature *T*_f_, at which the supercooled liquid structure was frozen, could encapsulate the effects of the average atomic spacing or structural heterogeneity on the energy state. However, the energy-matching method [22,23] cannot yield the *T*_f_ for some MGs lacking a stable supercooled liquid region in their calorimetric curves. Recently, Gao et al. [24,25] have proposed integrating the enthalpy change throughout the glass transition, defining it as the effective enthalpy Δ*H*_eff_. They suggest that Δ*H*_eff_ could effectively capture the rejuvenation of well-aged MGs. Consequently, the effective enthalpy Δ*H*_eff_ may serve as a rational parameter to bridge rejuvenation with the plasticity of MGs, but this expectation needs further experimental evidence.

In this paper, the structure and plasticity of typical Zr-based MGs are purposefully modulated by two different rejuvenation protocols: elastostatic compression and cycled twists. The plastic deformability of these rejuvenated samples is found to be linearly correlated with Δ*H*_eff_. Our results provide experimental evidence that the plastic yielding of MGs is similar to a stress-driven glass transition process. Structural analysis reveals that the decoupling of medium-range-order (MRO) structures contributes to higher Δ*H*_eff_, thus facilitating better plasticity.

## 2 Materials and methods

A typical Vit105 (Zr_52.5_Ti_5_Cu_17.9_Ni_14.6_Al_10_) MG was selected for this study due to its excellent glass-forming ability and broad range of rejuvenated states and associated plasticity [24,[26], [27], [28]]. Vit105 rods with a diameter *d* of 3 mm and a length *L* of 150 mm were fabricated via suction casting. Some of the rods were cut into 6-mm-height cylindrical samples for elastostatic compression. Using an Instron 8800 testing machine, the samples were compressed at 90% yield strength (∼1.8 GPa [26,29,30]) for 0.5, 1, 2, and 3 days, denoted as C0.5, C1, C2, and C3, respectively. Other as-cast samples were used for cycled twists at a CTTM502 twist-testing machine. The ends of these samples were fixed by clamps, allowing an effective twist length *L*_eff_ of 120 mm. The relative twist angle *φ* was controlled at 0.8π radian with a rate of 0.5π radian/s. The equivalent frequency of cycled twists was approximately 0.3 Hz. The maximum shear stress introduced by twists can be estimated by the following relation:

(1) $$\tau_{\text{max}}=\frac{Gd\varphi }{2L_{\text{eff}}},$$

where *G* ∼30 GPa is the shear modulus. The calculated *τ*_max_ is about 0.94 GPa, which is less than the shear yield strength (∼1 GPa [[31], [32], [33]]) of Zr-based MGs, thus avoiding the introduction of any mechanical failure. The loading-unloading cycle was repeated 100, 200, 400, and 600 times, and the obtained cycle-twisted samples were denoted as T100, T200, T400, and T600, respectively.

The amorphous nature of all samples (as-cast and deformed) was confirmed by the presence of typical diffuse scattering peaks in the XRD patterns, as shown in Fig. 1. Thermodynamic properties were examined by TA25 differential scanning calorimetry (DSC) and Quantum Design physical property measuring system (PPMS). To investigate the plastic deformability, all samples, as-cast and deformed, were fabricated into 6-mm-height cylinders for quasi-static compressions at a strain rate of 10^−4^ s^−1^. Three compression tests were performed on each sample to verify reproducibility. The repeated stress-strain curves are shown in Fig. S1. After compressions, the fracture morphologies were examined via FEI Sirion400NC scanning electron microscopy (SEM). Nanoindentation measurements were tested by Nano Indenter G200 with Berkovich diamond indenter, with a peak load of 300 mN and a loading rate of 5 mN/s The measurement layout is shown in Fig. S2a, with a total of 57 test points, including the center point of the cross-section and another 56 points selected at intervals of 190 µm along eight radial directions based on the center point. Finally, the atomic packing structures of all samples were investigated using synchrotron high-energy XRD at the Beijing synchrotron radiation facility.

**Fig. 1:**
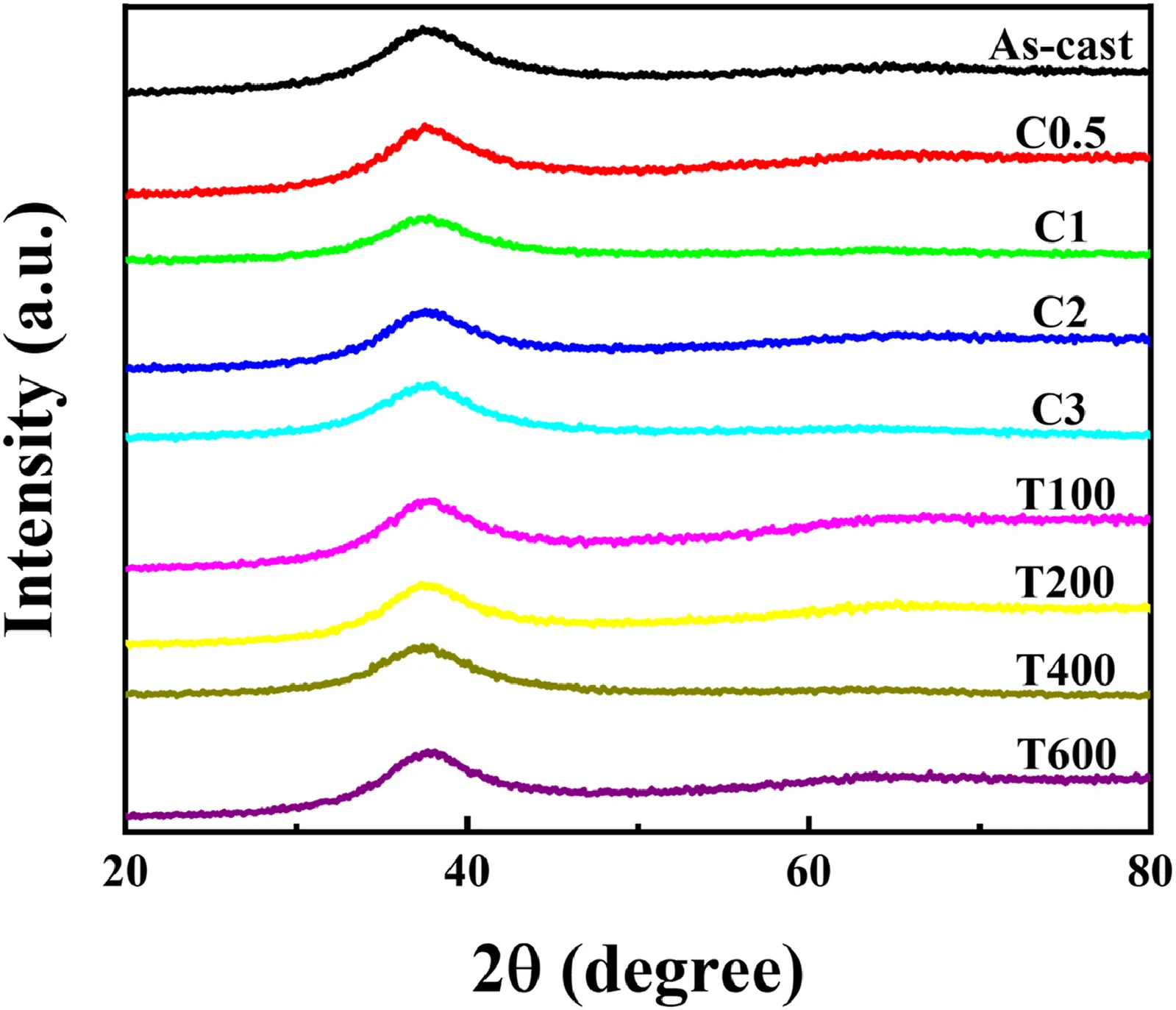
**XRD patterns of the as-cast, elastostatic-compressed, and cycle-twisted samples**.

## 3 Results and discussion

### 3.1 Thermodynamic properties

Fig. 2a shows the DSC curves of the as-cast, elastostatic-compressed, and cycle-twisted samples with the test temperature *T* from 450 K to 730 K. The DSC curves of all samples before heat release and after complete crystallization overlapped, demonstrating the stability of the baseline during the test and confirming the reliability of the experimental results. The black dashed line represents the crystallization baseline. The ∆*H*_exo_ is obtained by integrating the exothermic area below the baseline and the values are listed in Table 1. The ∆*H*_exo_ of the as-cast Vit105 MG is ∼0.394 kJ mol^−1^, which agrees with previous reports [24,34]. It is found that ∆*H*_exo_ values increase with increasing duration of elastostatic compression or number of twist cycles. This reflects the increase in free volume due to the cumulative anelastic deformation [[35], [36], [37]], indicating the rejuvenation of all deformed samples. Similar phenomena were also observed by Louzguine-Luzgin et al. [38] and Costa et al. [39]. They demonstrated that ∆*H*_exo_ is proportional to the unrecovered anelastic deformation in MGs. Moreover, both elastostatic compression and cycled twists reduce the glass transition overshoot of MGs, but the latter's effect is more significant. For example, the glass transition overshoot of T600 is lower than that of C3, despite they have similar ∆*H*_exo_ values. Previous studies [40,41] have suggested that the magnitude of glass transition overshoot is associated with the barrier of large-scale atomic rearrangement. This implies that the cycle-twisted samples may be more prone to glass transition than the elastostatic-compressed ones.

The structural rejuvenation of the samples was further characterized by their vibrational dynamics. It has been known that MGs usually manifest excessive vibrational modes beyond the Debye level at terahertz frequencies [42]. These excess vibrations result in an augmented specific heat capacity *C*_p_ at low temperatures, behaving as the boson peak (BP) [[43], [44], [45]]. For MGs, the low-temperature *C*_p_ comprises the phononic and electronic contributions. The electronic *C*_p_ is equal to *γ*_s_*T*, where the Sommerfield coefficient *γ*_s_ is determined by fitting *C*_p_ versus *T*^2^ below 8 K. The fitting processes are shown in Fig. S3. Consequently, BPs can be visualized in the plot of (*C*_p_‐*γ*_s_*T*)/*T*^3^ versus *T*, as shown in Fig. 2b The changes of BPs are enlarged in the inset. Their intensity values *H*_ph_ are listed in Table 1. Both elastostatic compression and cycled twists enhance BPs, and an approximately linear correlation between *H*_ph_ and ∆*H*_exo_ is shown in Fig. 1c by the red dashed line. The synchronization between ∆*H*_exo_ and *H*_ph_ is also found in our previous results [24,25], indicating the glass's rejuvenation. It is well accepted that the BP is related to the elastic heterogeneity of glasses on the nanoscale [21,46]. So, the observed positive *H*_ph_-∆*H*_exo_ correlation implies that glasses with higher ∆*H*_exo_ are structurally more heterogeneous. However, it is noted that the *H*_ph_ of T600 is higher than that of C3 despite their similar ∆*H*_exo_, showing a slight deviation from the red dashed line in Fig. 1c. This result is consistent with the lower glass transition overshoot of the T600 sample in Fig. 2a.

**Fig. 2:**
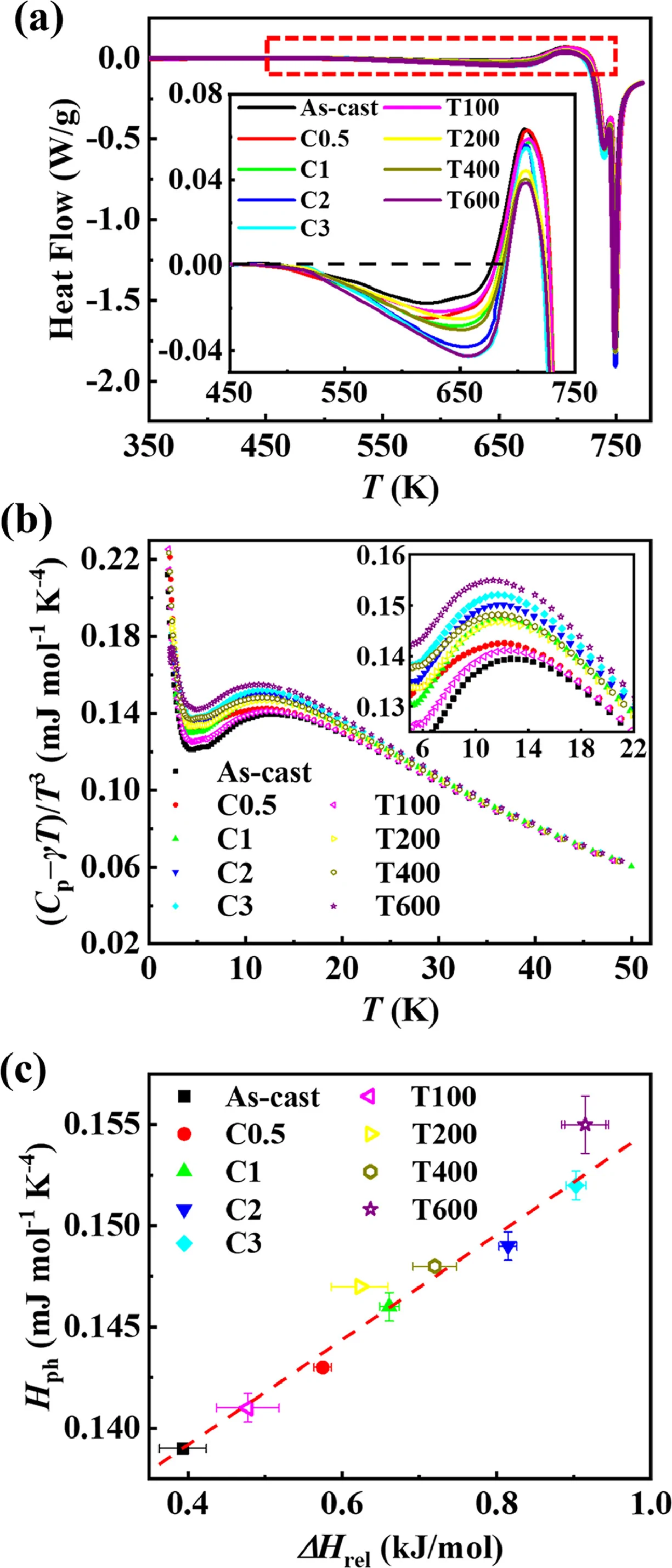
**Thermodynamic responses of the as-cast, elastostatic-compressed, and cycle-twisted samples.** (a) DSC curves of all samples. The inset shows the enlargement of the dashed box. The black dashed line represents the horizontal extension of the crystallization baseline. (b) Plots of (*C*_p_‐*γ*_s_*T*)/*T*^3^ versus *T* between 1.9 to 50 K. The changes in BP's intensity are magnified in the inset. (c) Correlation between *H*_ph_ and ∆*H*_exo_ for all samples. The red dashed line serves as a guide for the eye.

**Fig. 3:**
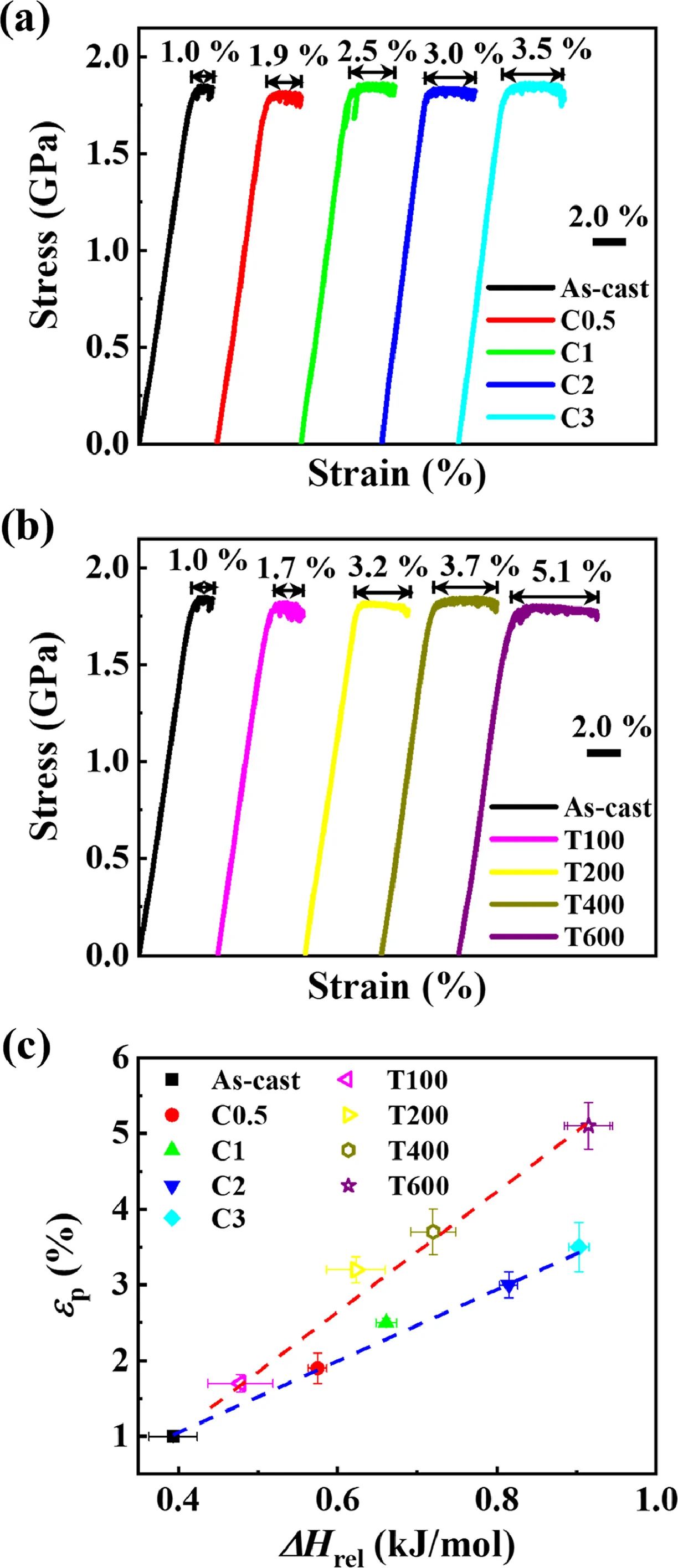
**Quasi-static compressive plasticity of all samples.** Stress-strain curves of (a) the as-cast and elastostatic-compressed samples, and (b) the as-cast and cycle-twisted samples. The plastic deformation segments of all samples are marked by double arrows. The plastic strain *ε*_p_ of each sample is directly labeled above the corresponding double arrow for easy reference. (c) Correlation between *ε*_p_ and ∆*H*_exo_ for as-cast and elastostatic-compressed samples (solid dots), and cycle-twisted samples (hollow dots). The dashed lines serve as a guide for the eye.

### 3.2 Quasi-static compression

Fig. 3a-b show the stress-strain curves of the as-cast, elastostatic-compressed, and cycle-twisted samples under the quasi-static compressions. The plastic deformation segments *ε*_p_ of all samples are marked by double arrows and the values are listed in Table 1. The *ε*_p_ of the as-cast sample is ∼1.0%, aligning with the value of the typical as-cast Vit105 MGs [26,29,30]. We find that the *ε*_p_ of both elastostatic-compressed and cycle-twisted MGs is enhanced compared to that of the as-cast one. We further plot the correlation between *ε*_p_ and ∆*H*_exo_, as shown in Fig. 3c. As-cast and elastostatic-compressed samples are represented by solid dots, while cycle-twisted samples are denoted by hollow dots. We observe two positive correlations between *ε*_p_ and ∆*H*_exo_: one for elastostatic-compressed samples (blue dashed line), the other for cycle-twisted samples (red dashed line). Under identical ∆*H*_exo_ conditions, the *ε*_p_ of the cycle-twisted samples surpasses that of elastostatic-compressed ones. A similar situation is expected for the *ε*_p_-*H*_ph_ correlation, considering a nearly linear relationship between *H*_ph_ and ∆*H*_exo_ (Fig. 2c). These results indicate that neither the system-averaged free volume nor the BP intensity is sufficient to evaluate the plasticity induced by rejuvenation. In other words, the rejuvenated MGs with the same ∆*H*_exo_ or *H*_ph_ possibly exhibit totally different plastic deformability.

**Fig. 4:**
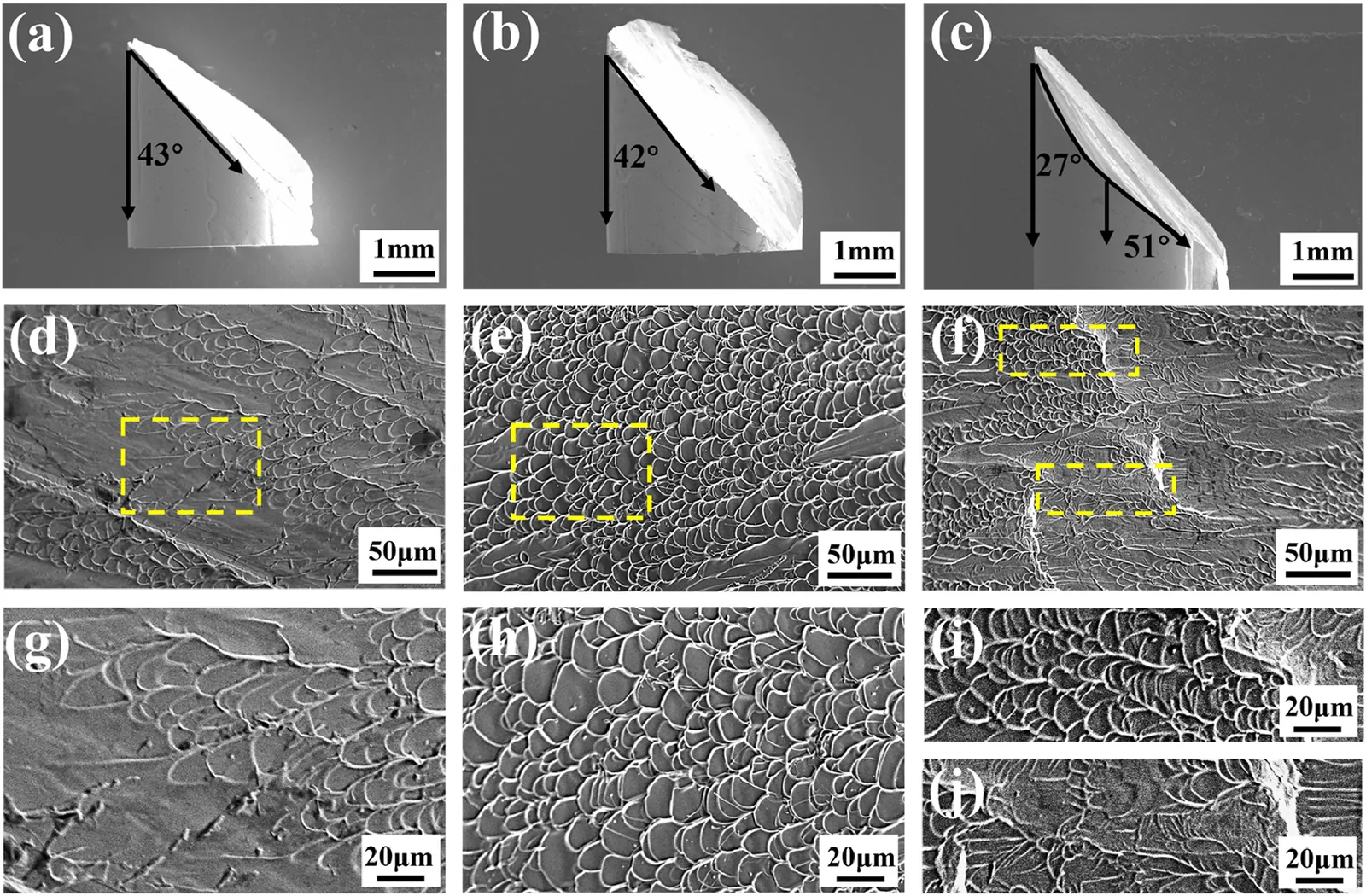
**Fracture characteristics after quasi-static compression.** The lateral views and sketches depicting fracture angles of (a) the as-cast, (b) C3, and (c) T600 samples. Fracture morphologies of (d) the as-cast, (e) C3, and (f) T600 samples. (g) and (h) show enlargements of the dashed boxes in (d) and (e), respectively. (i) and (j) show enlargements of the upper and lower dashed boxes in (f), respectively.

The fracture characteristics of samples are investigated to elucidate their deformation mechanism. The lateral views of three representative samples: as-cast, C3, and T600, are depicted in Fig. 4a-c, respectively. The fracture surfaces of the as-cast and C3 are straight, and their fracture angles are measured as 43° and 42°, respectively, indicative of typical compressive shear fracture modes [47,48]. In contrast, the fracture surface of the T600 exhibits a bent shape, with the fracture angle deflecting from the initial 27° to 51° The difference in fracture angle suggests a distinct fracture mode in the T600 sample compared to the as-cast and C3 samples. Moreover, the fracture-surface morphologies of the as-cast, C3, and T600 samples are depicted in Fig. 4d-f, respectively. Fig. 4g-h show the enlargement of the dashed boxes in Fig. 4d-e, respectively. Fig. 4i-j show the enlargement of the upper and lower dashed boxes in Fig. 4f, respectively. As depicted in Fig. 4 g, the fracture morphologies of the as-cast sample consist of vein-like patterns and intermittent smooth regions. For the C3 and T600 samples, the content and density of vein-like pattern regions are increased, as shown in Fig. 4h-i. Additionally, in the T600 sample, a river-like pattern is observed alongside the vein-like patterns, as magnified in Fig. 4j. Similar fracture characteristics can be found in other rejuvenated samples, as illustrated in Figs. S4–5.

During the propagation of shear bands, localized softening or melting results in the appearance of vein-like patterns which are typically associated with the soft regions of MGs. As depicted in Fig. 2a, the elastostatic compression increases the amount of free volume in MGs. This facilitates the formation of more vein-like patterns during shear band propagation, as evidenced in Fig. 4e and 4h Consequently, plasticity is enhanced in elastostatic-compressed samples. According to Ryu et al. [49], river-like patterns predominantly occur in the hard regions of MGs, impeding shear band propagation. In the fracture morphologies of cycle-twisted samples, both vein-like and river-like patterns are observed. This implies that the shear band encounters hard regions during its propagation, resulting in deflected propagation paths, as shown in Fig. 4c. Tang et al. [28] proposed that, due to its longer path, the deflected shear band propagation accommodates more plastic deformation than straight propagation. These two different modes of shear-band propagation are consistent with the observed plastic gap between the elastostatic-compressed and cycle-twisted samples. These results confirm that, in addition to averaged free volume, the structural heterogeneity associated with the arrangement of soft and hard zones benefits the global plasticity of MGs.

### 3.3 Correlation between effective enthalpy and plasticity

It becomes evident that the global plasticity of MGs is governed by large-scale structural rearrangements, which cannot be solely assessed by ∆*H*_exo_. From a thermodynamic standpoint, the stability of the glass structure is linked to both the structural exothermic enthalpy and endothermic glass transition overshoot. Gao et al. [24,25] demonstrated that the decreased stability of rejuvenated MGs is well captured by Δ*H*_eff_. As illustrated in Fig. 5a, Δ*H*_eff_ for Vit105 MGs, which quantifies the enthalpy change throughout the glass transition, can be calculated using the following relation:

(2) $$\begin{array}{c} \Delta H_{\text{eff}}=\int_{T_{1}}^{T_{2}}-C_{p}\;\text{dT,} \end{array}$$

where $T_{1}$ denotes the starting temperature of the exothermic area, and $T_{2}$ represents the ending temperature of the endothermic area. The calculation details are shown in Fig. S6. The values of Δ*H*_eff_ for all samples are summarized in Table 1. Both elastostatic compression and cycled twists enhance Δ*H*_eff_, and a linear correlation between *ε*_p_ and Δ*H*_eff_ is built for all rejuvenated samples, as guided by the red dashed line in Fig. 5b. This result suggests that the plasticity of MGs is influenced by both ∆*H*_exo_ and the endothermic glass transition overshoot, and Δ*H*_eff_ serves as a superior parameter for evaluating the plasticity of MGs compared to ∆*H*_exo_.

**Fig. 5:**
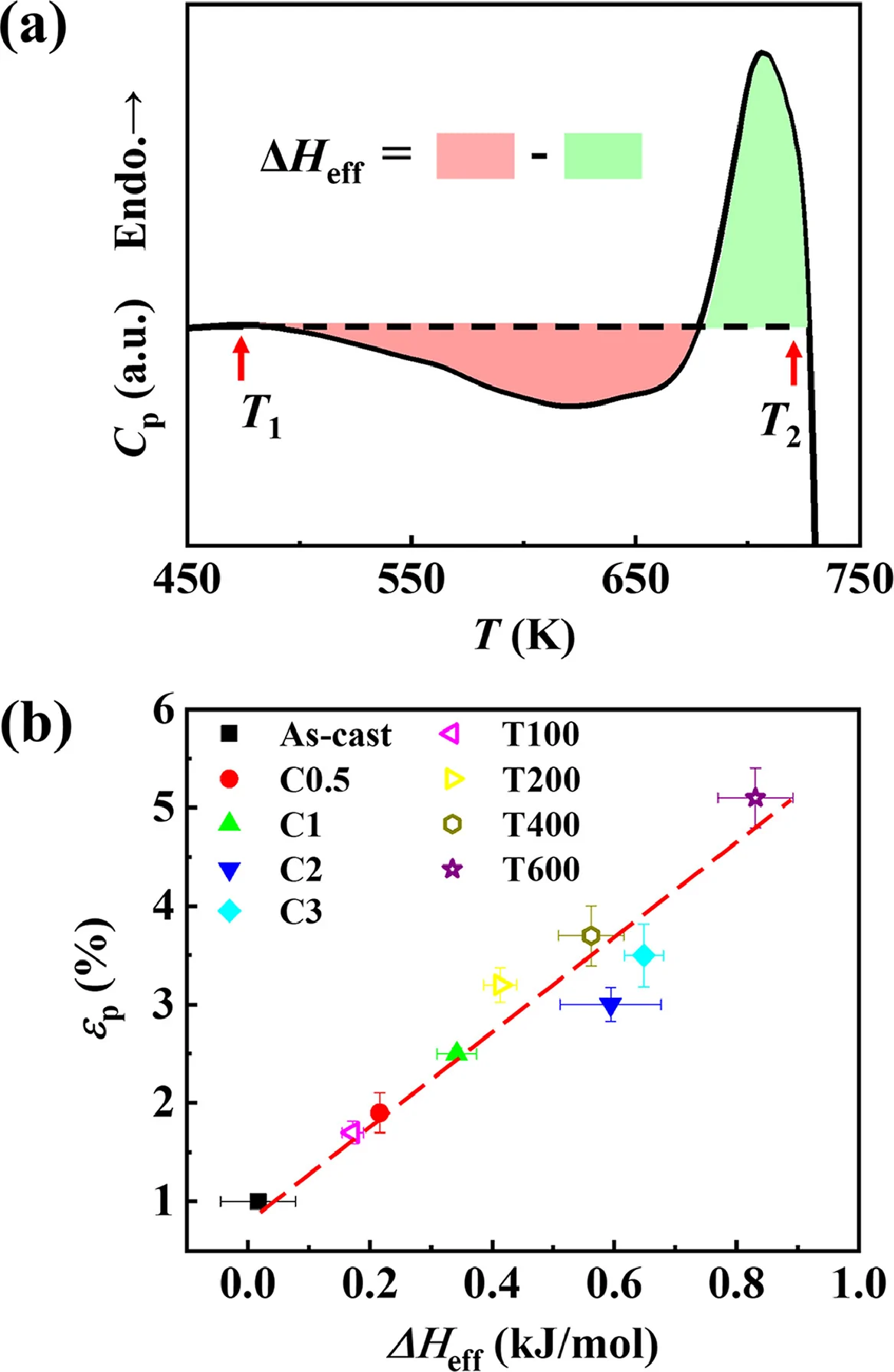
**(a) Schematic illustration for calculating effective enthalpy Δ*H*_eff_**. (b) Correlation between plastic strain *ε*_p_ and Δ*H*_eff_ for all rejuvenated samples. The black dashed line in (a) represents the horizontal extension of the crystallization baseline. A red dashed line in (b) is drawn as a guide for linear relationships.

**Table 1:** **Values of the exothermic enthalpy ∆*H*_exo_, BP intensity *H*_ph_, effective enthalpy Δ*H*_eff_, plastic strain *ε*_p_, position *q*_1_ of the first diffraction peak, and average hardness**$\bar{H}$**for all samples.**

Sample	∆*H*_exo_	*H*_ph_	Δ*H*_eff_	*ε*_p_	*q*_1_	$\bar{H}$
	(kJ mol^-1^)	(mJ mol^-1^ K^-4^)	(kJ mol^-1^)	(%)	(Å^-1^)	(GPa)
As-cast	0.394 ± 0.030	0.139 ± 0.001	0.017 ± 0.060	1.0 ± 0.057	2.652	6.619 ± 0.033
C0.5	0.575 ± 0.011	0.143 ± 0.001	0.216 ± 0.035	1.9 ± 0.264	2.646	6.555 ± 0.034
C1	0.661 ± 0.013	0.146 ± 0.002	0.342 ± 0.033	2.5 ± 0.200	2.644	6.531 ± 0.041
C2	0.815 ± 0.011	0.149 ± 0.002	0.594 ± 0.083	3.0 ± 0.057	2.642	6.468 ± 0.040
C3	0.903 ± 0.013	0.152 ± 0.002	0.648 ± 0.032	3.5 ± 0.264	2.638	6.461 ± 0.039
T100	0.478 ± 0.041	0.141 ± 0.002	0.172 ± 0.018	1.7 ± 0.153	2.647	6.578 ± 0.047
T200	0.623 ± 0.037	0.147 ± 0.001	0.413 ± 0.027	3.2 ± 0.200	2.646	6.521 ± 0.051
T400	0.720 ± 0.028	0.148 ± 0.001	0.562 ± 0.053	3.7 ± 0.400	2.644	6.503 ± 0.050
T600	0.915 ± 0.027	0.155 ± 0.003	0.831 ± 0.062	5.1 ± 0.351	2.638	6.461 ± 0.062

Analogous to the glass transition overshoot in calorimetric curves, MGs generally exhibit stress overshoot before steady-state plastic flow during high-temperature uniform deformation [24,25,27]. For a system with insufficient free volume to match the final flow state, the stress overshoot results from the need for shear stresses higher than the flow stress to activate the STs [50]. Thus, the magnitude of stress overshoot evaluates the amount of free volume required for the system to reach the plastic flow. Moreover, molecular dynamics simulations [51,52] demonstrate that the emergence of the shear band and plastic flow is a consequence of stress-induced glass transition because of their simultaneous changes in viscosity, enthalpy and configurational temperature. Based on this framework, we argue that the creating free volume required for the shear-band emergence can be evaluated by the endothermic glass transition overshoot. As the ∆*H*_eff_ increases, the internal structure and energy state of the systems become closer to the liquid equilibrium state, which means that the MGs need less creating free volume to undergo stress-induced glass transition, thus enhancing plasticity.

### 3.4 Structural properties

The structural softening induced by rejuvenation can be reflected by mechanical hardness. Typical nanoindentation load- and unload-depth curves for all samples are shown in Fig. S2b. The mechanical hardness *H* is determined using Meyer's method [53], and the section-averaged values $\bar{H}$ for samples are listed in Table 1. A negative linear correlation between *H* and ∆*H*_exo_ is observed in Fig. 6a, which is consistent with previous reports [8,54]. Notably, the error bars of the cycle-twisted sample are more pronounced than those of the elastostatic-compressed sample. The variation in hardness along the radial distance from the cross-section center for three representative samples—namely, as-cast, C3, and T100—are presented in Fig. 6b. The $\bar{H}$ of the C3 and T100 samples is the same and lower than that of the cast samples. Despite having the same $\bar{H}$, the T100 sample exhibits a harder core, and a softer shell compared to the C3 sample. Similar phenomena can be observed in other rejuvenated samples, as shown in Fig. S2c. The structural differences between elastostatic-compressed and cycle-twisted samples primarily arise from the different stress distributions they are subjected to. Elastostatic-compressed samples were subjected to uniformly distributed compressive stresses, resulting in relatively uniform structural rejuvenation. In contrast, cycle-twisted samples are subjected to radially varying torsional stresses, with the highest stress in the outermost regions and the lowest in the center. During the cycled twist, the atomic rearrangements in the outer region are more easily activated, thereby accumulating more anelastic deformation. The resulting gradient structure leads to the better plasticity of the cycle-twisted samples compared to the elastostatic-compressed ones under similar ∆*H*_exo_.

**Fig. 6:**
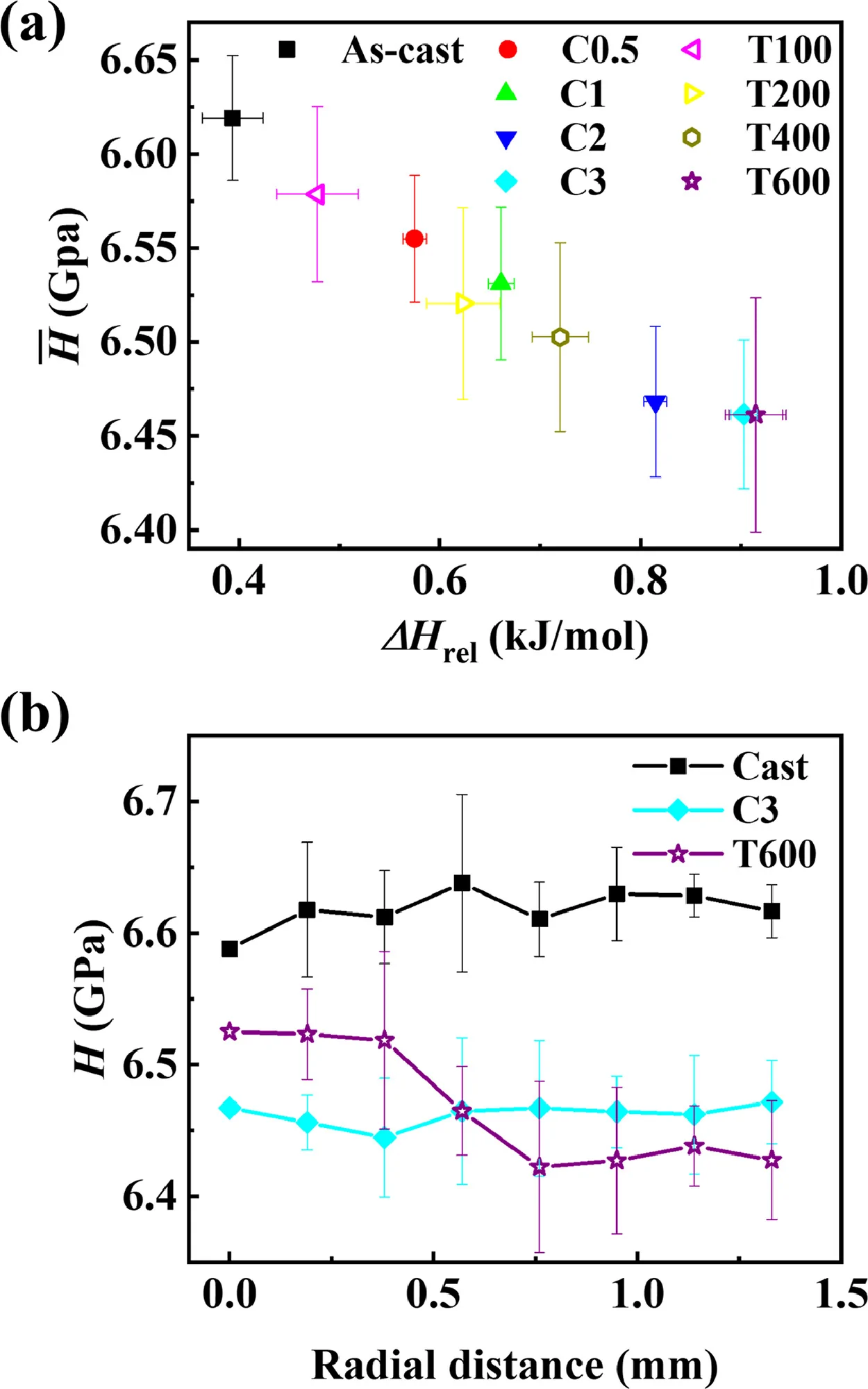
**Nanoindentation responses.** (a) Correlation between section-averaged hardness $\bar{H}$ and exothermic enthalpy ∆*H*_exo_ for all samples. (b) Variation in mechanical hardness *H* along the radial distance from the cross-section center for three representative samples—namely, as-cast, C3, and T100.

At last, the structural changes of all samples are meticulously analyzed by the synchrotron high-energy XRD. Fig. 7a displays the diffraction intensity curves *I*(*q*) of all samples. The inset provides a close-up view of the first peaks. Their positions *q*_1_ are listed in Table 1. As depicted in Fig. 7b, the *q*_1_ exhibits a linear decrease with the increased ∆*H*_exo_, suggesting that higher free volume existing in glasses correspond to larger average atomic spacing [27,55]. Since C3 and T600 samples have similar ∆*H*_exo_, their *q*_1_ are approximately equal, indicating that they have similar average atomic spacing. This suggests that the gap in their plasticity is mainly caused by different atomic topological rearrangements.

**Fig. 7:**
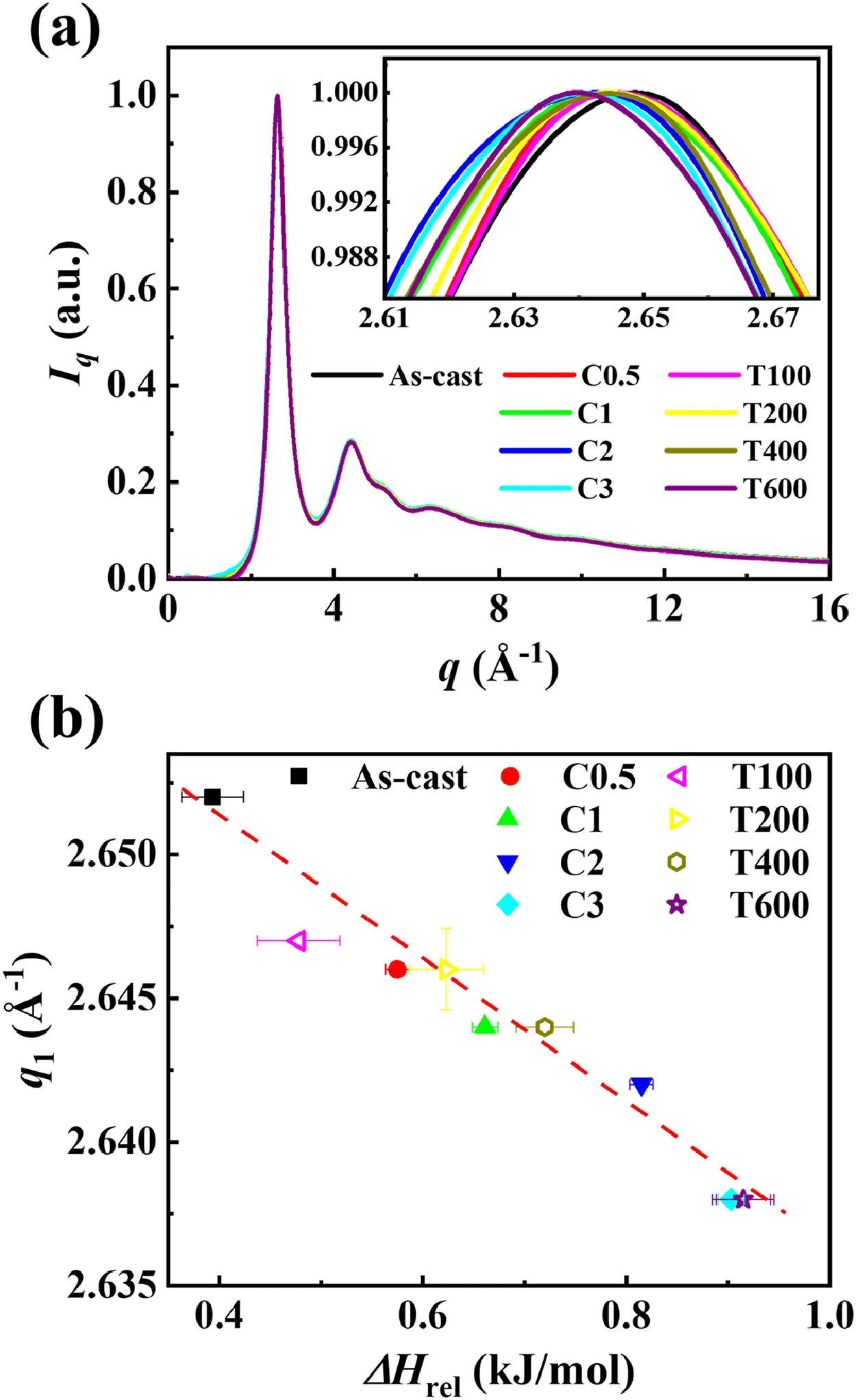
**High-energy XRD analysis of all samples.** (a) Diffraction intensity curves *I*(*q*). The inset provides a magnified view of the upper portion of the first peaks. (b) Correlation between the position of the first peak and exothermic enthalpy for all samples.

To further study the evolution of atomic packing topology, the radial distribution function (RDF), denoted as *g*(*r*), is obtained by Fourier transformation of I(q), as shown in Fig. 8a. Fig. 8b-c display enlarged views of the first and second RDF peaks for the as-cast and elastostatic-compressed samples, respectively. With increasing duration of elastostatic compression, the height of the first RDF peak gradually decreases, indicating a reduction in short-range order (SRO) content [56,57] and the structural rejuvenation of MGs. Besides, no peak splitting is observed for any peaks, suggesting that the rejuvenation induced by elastostatic compression primarily entails an expansion of the average atomic spacing rather than a reconstruction of the atomic configuration. Fig. 8d-e present enlarged views of the first and second RDF peaks for the as-cast and cycle-twisted samples, respectively. In addition to the decrease in the first RDF peak, the second RDF peak gradually transitions from a single peak of the as-cast state to double distinct peaks under the influence of cycled twists. Similar splitting phenomena were also observed in our recent work [58], where the Vit105 MG underwent cycled shock compressions. These results clearly indicate that cycled twists not only increase the average atomic spacing but also reconstruct the MRO structures.

**Fig. 8:**
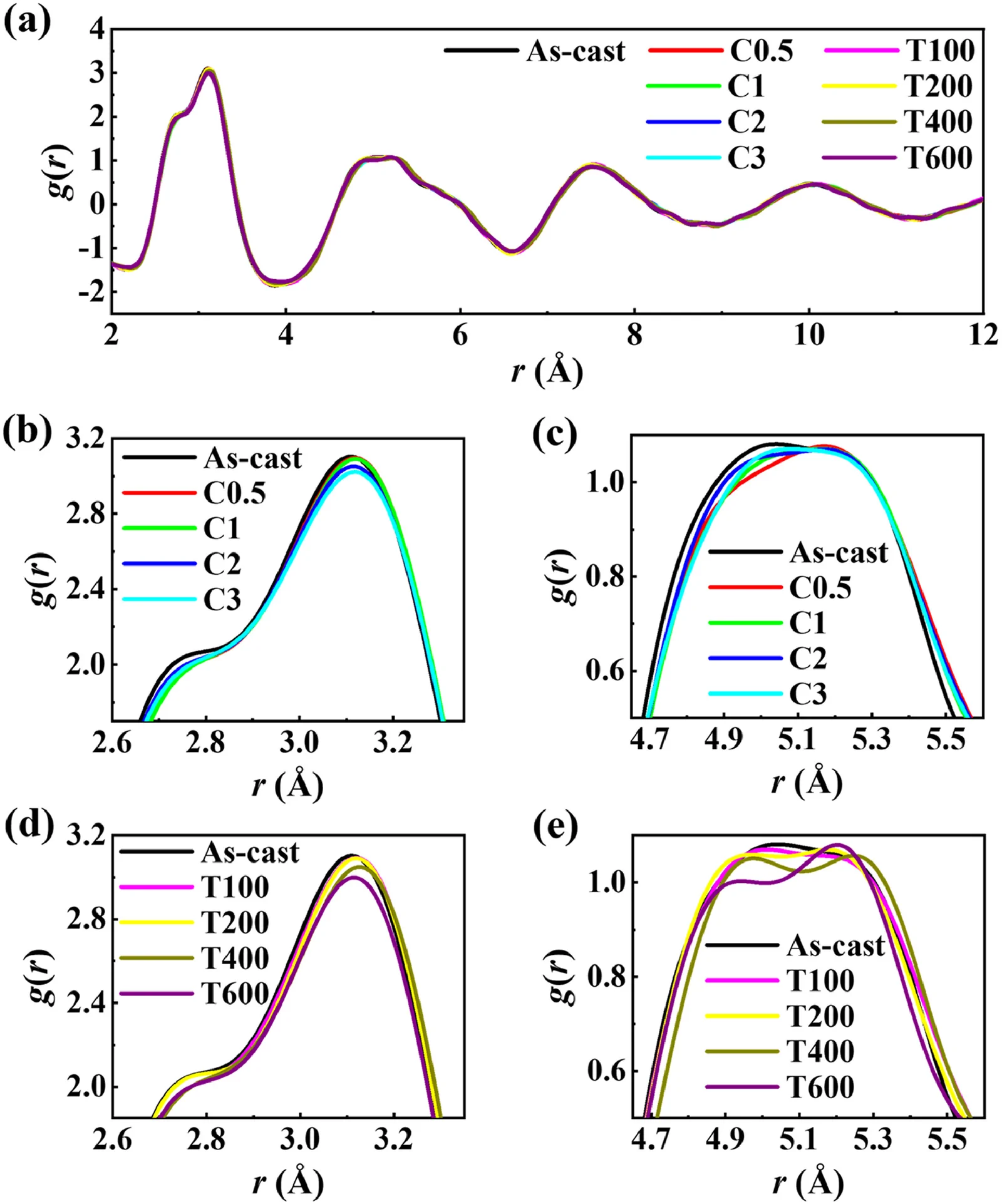
**Radial distribution functions of all samples.** (a) Overall trend of RDF curves. Enlarged views of (b) the first peak and (c) the second peak of RDFs for as-cast and elastostatic-compressed samples. Enlarged views of (d) the first peak and (e) the second peak of RDFs for as-cast and cycle-twisted samples.

Fig. 9 provides an enlarged view of the second RDF peak for three representative samples: the as-cast, C3 and T600. The ordinate and abscissa axes are normalized by the height *g*(*r*_c_) and position *r*_c_ of the first RDF peak of the as-cast sample, respectively. In the C3 sample, elastostatic compression results in a slight reduction in the height of the second RDF peak, with no discernible change in shape. In contrast, the second RDF peak of T600 sample splits into two sub-peaks located around 1.57 *r*_c_ and 1.67 *r*_c_ after cycled twists. The left sub-peak corresponds to interpenetrating MROs, whereas the right sub-peak corresponds to the face-sharing MROs [[58], [59], [60]]. As depicted in Fig. 9, cycled twists diminish the presence of interpenetrating MROs while augmenting face-sharing MROs. We believe that this decoupling of the MROs leads to higher Δ*H*_eff_ as well as better plasticity of the cycle-twisted samples. As MROs are intrinsic to glasses [[59], [60], [61]], it can be reasonably supposed that a general correlation between plastic deformability and effective enthalpy is valid in other MG systems.

**Fig. 9:**
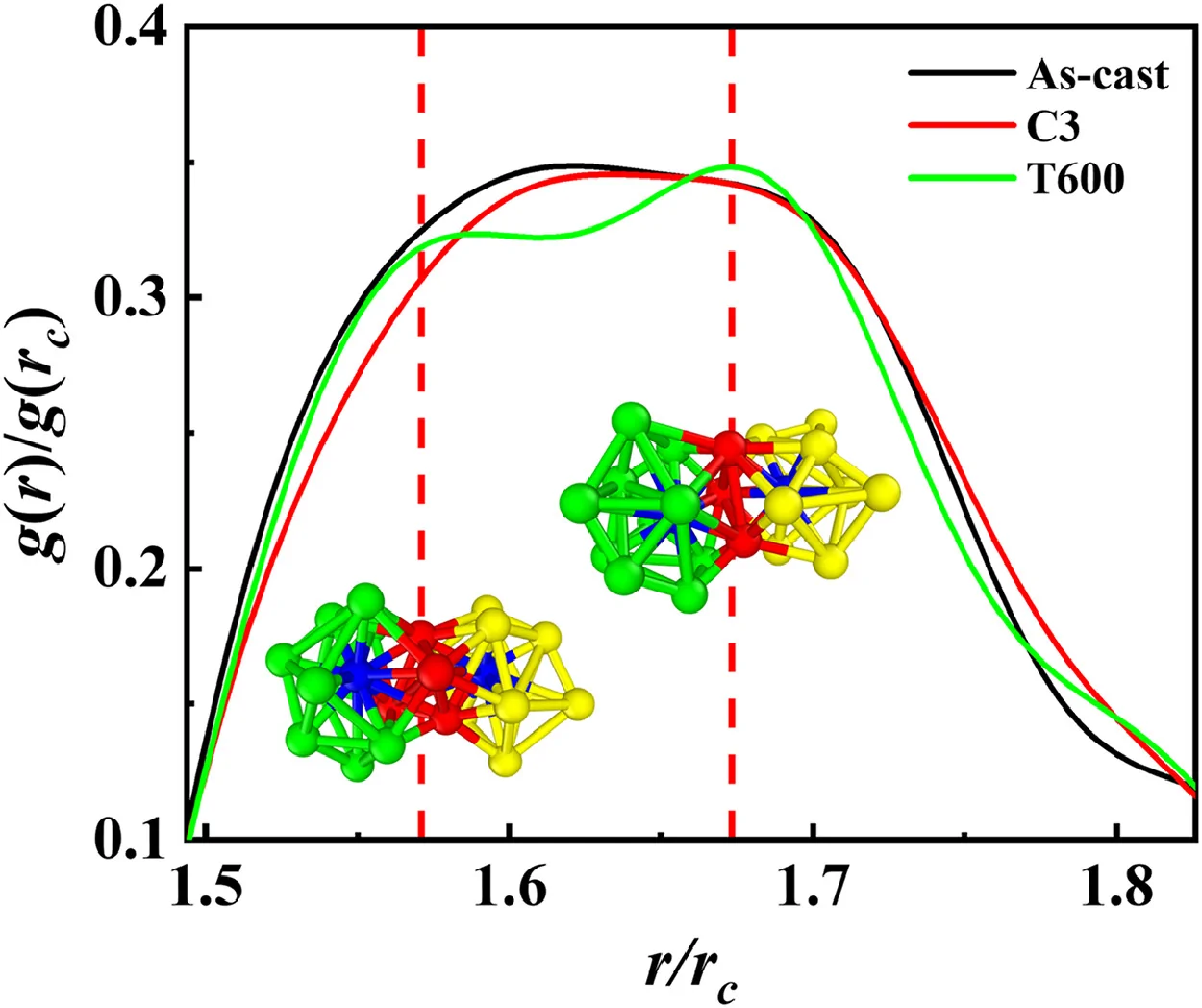
**Enlarged view of the second RDF peak for as-cast, C3 and T600 samples.** The ordinate and abscissa axes are scaled by the height *g*(*r*_c_) and position *r*_c_ of the first RDF peak of the as-cast sample, respectively. Vertical dashed lines denote the positions of distinct MRO types, including interpenetrating (1.57 *r*_1_) and face-sharing (1.66 *r*_1_). Insets provide schematics illustrating the respective MRO types. Blue spheres represent two central atoms acting as second neighbors to each other. Green and yellow spheres denote atoms within left and right atomic clusters, respectively, while red spheres represent shared atoms between two atomic clusters.

## 4 Conclusion

In this work, taking the Vit105 as a model material, we provide experimental evidence that the effective enthalpy can quantitatively evaluate the global plasticity of MGs that are rejuvenated by different methods (elastostatic compression and cycled twists). Either exothermic enthalpy before glass transition or BP intensity is only applicable to single-path-rejuvenated plasticity. With quasi-static compressions, we observe that the cycle-twisted glasses exhibit superior plastic deformation compared to the elastostatic-compressed ones. The two-path rejuvenated MGs present very different shear band modes and fracture morphologies. With nanoindentations, we also observe a more heterogeneous hardness distribution in the cycle-twisted glasses. Synchrotron high-energy XRD further reveals that both cycled twists and elastostatic compression increase the average atomic spacing, but simultaneously the former induces the unstable reconstruction of MROs. These results substantiate the idea that the global plasticity of MGs depends on not only the atomic free volume but also its spatial distribution beyond the atomic scale. Fortunately, the effective enthalpy defined here as a thermodynamic parameter can capture the two factors. A reasonable explanation is that plastic yield and glass transition possibly share similar structural rearrangements in MGs. Our work also provides new insight into designing MGs with excellent plasticity by rejuvenating them through the synchronous introduction of free volume and structural heterogeneity.

## Declaration of competing interest

The authors declare that they have no conflicts of interest in this work.

## Appendix Supplementary materials

Image, application 1
