# Supplementary material for: Evaluating plasticity of rejuvenated metallic glasses by effective enthalpy
Source: Fundam Res. 2025 Mar 25;6(5):2848–55. doi: 10.1016/j.fmre.2025.03.008 (PMC13644414; doi:10.1016/j.fmre.2025.03.008)
Supplement: Supplementary file 1 [file mmc1.docx]

**Supplemental Material**

**“Evaluating plasticity of** **rejuvenated metallic glasses by effective enthalpy”**

Cheng Yang^a,b^, Hong-Bo Zhou^a^, Jun Duan^a^, Song-Lin Cai^a^, Gan Ding^a^, Bing-Bing Zhang^d^, Cai-Juan Shi^d^, Lan-Hong Dai^a,b^, Gerhard Wilde^c^, Min-Qiang Jiang^a,b,^^[1]^

^a^ *State Key Laboratory of Nonlinear Mechanics, Institute of Mechanics, Chinese Academy of Sciences, Beijing 100190, China*

^b^ *School of Engineering Science, University of Chinese Academy of Sciences, Beijing 101408, China*

^c^ *Institute of Materials Physics, University of Münster, Münster 48149, Germany*

^d^ *Beijing Synchrotron Radiation Facility, Institute of High Energy Physics, Chinese Academy of Sciences, Beijing 100049, China*

# 1. Quasi-static compression tests

Quasi-static compression tests of all samples, as-cast and deformed, were conducted at room temperature using an Instron 8800 testing machine, with a strain rate of 10^-4^ s^-1^. Three compression tests were performed on each sample to verify reproducibility. The repeated stress-strain curves are shown in Fig. S1.

**Fig. S1. Stress-strain curves for all samples across the three repetitions.**

# 2. Nanoindentation responses

Each sample was subjected to nanoindentation measurements following the same protocol. The layout is shown in Fig. S2a, with a total of 57 points, including the center point of the cross-section and another 56 points selected at intervals of 190 μm along eight radial directions based on the center point. Typical nanoindentation load- and unload-depth curves of the as-cast, elastostatic-compressed, and cycle-twisted samples are shown in Fig. S2b. The mechanical hardness is determined using Meyer's method. Fig. S2c shows the variation in mechanical hardness *H* along the radial distance from the cross-section center of all samples. The cycle-twisted sample exhibits a gradient structure compared to the elastostatic-compressed sample, resulting in a higher rejuvenation of the former at similar free volume content.

**Fig. S2. Nanoindentation results.** (a) Schematic illustration of measurement layout. (b) Typical nanoindentation load- and unload-depth curves of the as-cast, elastostatic-compressed, and cycle-twisted samples. (c) Variation in mechanical hardness *H* along the radial distance from the cross-section center of the as-cast, elastostatic-compressed, and cycle-twisted samples.

# 3. Determination of the electronic *C*_p_ contribution

For MGs, the low-temperature *C*_p_ comprises the phononic and electronic contributions. The electronic *C*_p_ is equal to *γ*_s_*T*. To estimate the Sommerfield coefficient *γ*_s_, it is necessary to fit the formula *C*_p_/*T*=*βT*^2^+*γ*_s_ in the temperature range below 8 K. This process is shown in Fig. S3.

**Fig. S3. Determination of the electronic *C*_p_ contribution.** (a) Low temperature *C*_p_ of all samples in the temperature range from 1.9 K to 50 K. (b)-(j) Plots of *C*_p_/*T* vs *T*^2^. The dashed line represents the linear fit of *C*_p_/*T*=*βT*^2^+*γ*_s_.

# 4. Fracture characteristics

Figs. S4-5 show the lateral and fracture surfaces of the elastostatic-compressed and cycle-twisted sample, respectively. The shear band of the elastostatic-compressed sample mainly propagates along one direction with a fracture angle of ~42°. The enhanced plasticity of the elastostatic-compressed sample is mainly reflected in the denser vein-like pattern on the fracture surface. The propagation of shear band is deflected in the cycle-twisted sample, which leads to its two distinct shear band propagation modes: vein-like and river-like patterns. The deflected propagation of shear band improves plasticity more effectively than propagation in a single direction.

**Fig. S4. Fracture characteristics of elastostatic-compressed sample.** The lateral views and sketches depicting fracture angles of (a) the C0.5, (b) C1, and (c) C2 samples. Fracture morphologies of (d) the C0.5, (e) C1, and (f) C2 samples. (g)-(i) show enlargements of the dashed boxes in (d)-(f), respectively.

**Fig. S5. Fracture characteristics of cycle-twisted sample.** The lateral views and sketches depicting fracture angles of (a) the T100, (b) T200, and (c) T400 samples. Fracture morphologies of (d) the T100, (e) T200, and (f) T400 samples. (g)-(i) show enlargements of the upper dashed boxes in (d)-(f), respectively. (j)-(l) show enlargements of the lower dashed boxes in (d)-(f), respectively.

# 5. Calculation of effective enthalpy

Effective enthalpy quantifies the enthalpy change throughout the glass transition and can be calculated using the following relation:

${\text{∆}\text{H}}_{\text{eff}}\text{=}\int_{\text{T}_{\text{1}}}^{\text{T}_{\text{2}}} \text{̵}\text{C}_{\text{p}}\text{ d}\text{T}$,	(1)

where *T*_1_ denotes the starting temperature of the exothermic area, and *T*_2_ represents the ending temperature of the endothermic area. The integration results for all samples are shown in Fig. S6.

**Fig. S6. Calculation of effective enthalpy Δ*H*_eff_ for all samples.**

1. Corresponding author.

   *E-mail address*: mqjiang@imech.ac.cn (M.-Q. Jiang).
